# Wicked conflict: Using wicked problem thinking for holistic management of conservation conflict

**DOI:** 10.1111/conl.12460

**Published:** 2018-04-24

**Authors:** Tom H.E. Mason, Chris R.J. Pollard, Deepthi Chimalakonda, Angela M. Guerrero, Catherine Kerr‐Smith, Sergio A.G. Milheiras, Michaela Roberts, Paul R. Ngafack, Nils Bunnefeld

**Affiliations:** ^1^ Biological and Environmental Sciences, Faculty of Natural Sciences University of Stirling Stirling FK9 4LA United Kingdom; ^2^ Conservation Ecology Group, Department of Biosciences Durham University South Road Durham DH1 3LE United Kingdom; ^3^ Department of Biological Sciences National University of Singapore 14 Science Drive 4 Singapore 117543; ^4^ School of Biological Sciences The University of Queensland Brisbane Queensland 4072 Australia; ^5^ Department of Security and Crime Science University College London 35 Tavistock Square London WC1H 9EZ United Kingdom; ^6^ Centre for Biodiversity & Environment Research (CBER), Department of Genetics, Evolution and Environment University College London London WC1E 6BT United Kingdom; ^7^ School of Geography & Sustainable Development, Irvine Building University of St Andrews North Street St Andrews KY16 9AL United Kingdom; ^8^ African Marine Mammal Conservation Organization PO Box 908 Edea Cameroon

**Keywords:** adaptive management, coexistence, comanagement, complex systems, conservation conflict, human‐wildlife conflict, resilience, structured decision‐making, uncertainty, wicked problems

## Abstract

Conservation conflict is widespread, damaging, and has proved difficult to manage using conventional conservation approaches. Conflicts are often “wicked problems,” lacking clear solutions due to divergent values of stakeholders, and being embedded within wickedly complex environments. Drawing on the concept of wicked environmental problems could lead to management strategies better suited to tackling conflict. However, it is unclear whether managers are embracing ideas from the wicked problems concept. There is currently a lack of guidance for applying strategies to tackle particular wicked problems, such as conservation conflict. We explored the suitability of wicked problems‐inspired management, using eight contemporary conflict case studies. Conservation conflict was managed predominantly using conventional approaches suited to tackling single objectives in simple environments, rather than balancing competing objectives in complex environments. To deal with different characteristics of wickedness, we recommend that managers develop strategies combining distributed decision‐making, diverse opinions, pattern‐based predictions, trade‐off‐based objectives, and reporting of failures. Recent advances in conservation conflict research have focused on improving interactions among stakeholders. We believe that such stakeholder‐focused approaches would dovetail with the whole‐system focus of a wicked problems framework, allowing conservationists to move toward a holistic strategy for managing conservation conflict.

## 1 INTRODUCTION

Conflicts over natural resources and conservation are widespread globally (Redpath, Gutiérrez, Wood, & Young, 2015), and can be highly damaging to UN Sustainable Development Goals such as biodiversity and food security (D'Harcourt, Ratnayake, & Kim, 2017). Conservation conflicts occur when individuals or groups have differing objectives regarding biodiversity management and one party is perceived to assert its actions at the expense of others (Redpath et al., 2013). They invariably involve interacting ecological, economic, and sociopolitical elements, with dynamic relationships driven by the attitudes, values, and power of the associated actors (Bunnefeld, Nicholson, & Milner‐Gulland, 2017). This complexity distinguishes conservation conflicts from the more straightforward problem of biodiversity impacts (Young et al., 2010) and has led to conservation conflicts being identified as “wicked problems” (Parrott, 2017; Redpath et al., 2015): intractable problems embedded in complex systems that are difficult to define and lack clear solutions (Rittel & Webber, 1973). Despite often being highly complex, conflicts tend to be treated as conventional, cause‐effect problems. Approaching conflicts instead as complex, nuanced problems could aid in identifying positive ways forward for their management (Young et al., 2010).

Conservation conflicts appear to meet many of the characteristics of wicked problems, which are defined by a set of traits relating to stakeholders and the wider system (Balint, Stewart, & Desai, 2011; Rittel & Webber, 1973). Differences in stakeholder values are central to wicked problems, and are considered one of the roots of conservation conflict (Redpath et al., 2013). From this, three other stakeholder characteristics emerge: differences in problem statements, objectives, and tactics. Wicked problems also tend to involve actors with varying levels of power, a feature that is ubiquitous across conservation conflicts due to stakeholders frequently varying greatly in number, wealth, and influence (Raik, Wilson, & Decker, 2008). Conservation conflicts are embedded in wickedly dynamic and uncertain socioecological systems, comprising nonlinear dynamics, multiple feedback loops, and high levels of scientific, political, and administrative uncertainty (Liu et al., 2007).

Conservation scientists have argued that approaching conservation challenges as wicked problems could lead to improved results (DeFries & Nagendra, 2017; Game, Meijaard, Sheil, & McDonald‐Madden, 2014), including for conservation conflict specifically (Redpath et al., 2013). However, it is unclear if or how managers are using wicked problem thinking to manage conservation conflicts. Furthermore, while types of management intervention suited to wickedness have been proposed (DeFries & Nagendra, 2017; Game et al., 2014), there is a lack of guidance for holistically tackling the varied features of wickedness within conservation problems such as conflict. Here, we explore these unknowns for eight conservation conflicts spanning five continents by evaluating the current and potential application of management approaches consistent with wicked problem thinking (hereafter “wicked approaches”).

## 2 APPLYING WICKED APPROACHES TO TACKLE CONSERVATION CONFLICT

**Figure 1:**
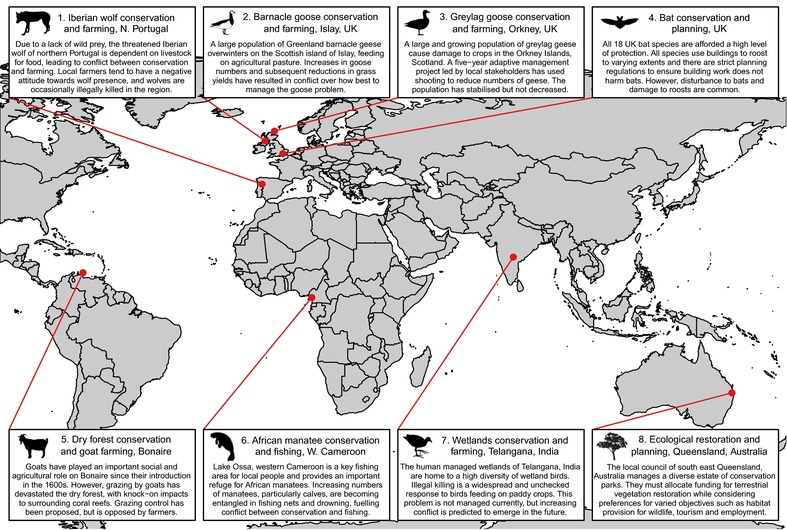
Map and descriptions of eight case studies of conservation conflict

Eight early‐career conservation researchers, each investigating a particular case study of conservation conflict, met at the “Interdisciplinary Conservation Network” workshop at the University of Oxford in June 2016. The case studies come from across the globe (Africa, Asia, Australia, the Caribbean, and Europe) and vary substantially in their environment (agricultural, freshwater, montane, tropical forest, urban, and wetland), spatial scale (from local districts to countries), historical development (from emerging to well established), and the taxa involved (mammals, birds, and plants) (Figure 1). The drivers of these conflicts are varied, for instance, being caused by disagreements among stakeholders over the appropriate size of wildlife populations, the efficacy or ethics of management options, and the importance of conservation objectives (see Table S1 for full details). All case studies met the majority of wicked problem characteristics outlined by Rittel and Webber (1973) (Table S2).

**Figure 2:**
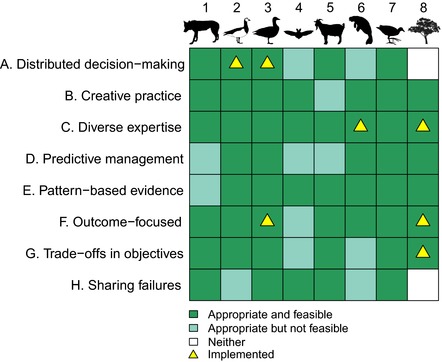
The appropriateness, feasibility, and implementation of wicked approaches, at the time of our analysis, for eight case studies of conservation conflict, represented by symbols (1. Iberian wolf conservation and farming, 2. Barnacle goose conservation and farming, 3. Greylag goose conservation and farming, 4. Bat conservation and planning, 5. Dry forest conservation and goat farming, 6. African manatee conservation and fishing, 7. Wetlands conservation and farming, and 8. Ecological restoration and planning; see Figure 1)

Researchers first identified whether conventional or wicked approaches were currently being implemented in their case study, using a set of corresponding conventional and wicked approaches adapted from Game et al. (2014) (Table 1). We found that conventional management approaches were widespread across the case studies, with only a small number of wicked approaches being applied currently (Figure 2). Second, researchers explored whether wicked approaches would potentially be appropriate and feasible for their case study, by assessing the following criteria based on their knowledge and experience:

1. Appropriateness: Would the wicked approach be likely to reduce levels of conflict among stakeholders more strongly than the corresponding conventional approach?
2. Feasibility: Would it be possible to implement the wicked approach over the next 5 years, assuming initial funding and political support at current levels?

**Table 1:** Corresponding conventional and wicked problem inspired management approaches, adapted from Game et al. (2014)

	Conventional	Wicked
**A**	*Top‐down decision making*	*Distributed decision‐making*
	Management decisions are made in a top‐down process	Management decisions are contributed to by different actors and organizations
**B**	*Standard practice*	*Creative practice*
	Standard management practices, applied elsewhere for other problems, are used	Creative management practices, suited to the specific problem, are developed
**C**	*Restricted expertise*	*Diverse expertise*
	Management is guided by restricted expertise	Management is guided by diverse expertise
**D**	*Passive management*	*Predictive management*
	Management interventions are adapted over time as the system is altered	Management interventions are adapted iteratively, following scenario‐based predictions
**E**	*Conventional evidence*	*Pattern‐based evidence*
	Management is informed by evidence from single processes	Management is informed by pattern recurrence in complex, interactive processes
**F**	*Strategy‐focused*	*Outcome‐focused*
	The type of management strategy that can achieve objectives is focused on	Objectives are focused on, allowing flexibility in strategy
**G**	*Objective success*	*Trade‐offs in objectives*
	Clear measures of management success are used	Trade‐offs in management success are acknowledged
**H**	*Avoid sharing failures*	*Sharing failures*
	Management failures are not shared with stakeholders	Management failures are shared transparently with stakeholders

We identified that wicked approaches would be widely appropriate across the case studies, though would not be feasible in some instances (Figure 2). Based on these findings, we report five key themes from which lessons can be learnt for achieving holistic management of conflict. These themes unite a variety of existing concepts and methods from different disciplines, including adaptive management (e.g., Bunnefeld et al., 2017), the resilience approach (e.g., Folke, Biggs, Norström, Reyers, & Rockström, 2016), collaborative governance (e.g., Bodin, 2017), and structured decision‐making (e.g., Guerrero et al., 2017). While adaptive management and resilience stem mostly from the natural sciences, collaborative governance, and structured decision‐making are rooted largely in the social and political sciences. Wicked approaches unify and bridge these different disciplines, providing a transdisciplinary framework for managing complex problems more holistically. Our themes tackle the varied properties of wicked problems, ranging from incorporating divergent values of stakeholders into objective setting to predicting management effectiveness under uncertainty. We identify outstanding research topics emerging from these themes that require systematic study (Table 2).

**Table 2:** Emergent research topics from wicked problems themes, requiring further study

Wicked theme	Emergent topics in conservation conflict research
i. Distributed decision‐making	‐ Relative performance of management under distributed and top‐down systems ‐ Exploration of spatiotemporal mismatch between governance and environment
ii. Diverse opinions and creativity	‐ Impacts of diverse knowledge on management
iii. Pattern‐based evidence and predictive management	‐ Utility of different types of pattern‐based evidence ‐ Relative performance of active and passive adaptive management ‐ Reliance of adaptive management on adaptive governance
iv. Trade‐off‐based objectives	‐ Development of optimizable trade‐off‐based objectives ‐ Relative performance of skewed versus trade‐off‐based objectives
v. Sharing failures	‐ Exploration of constraints to sharing failures ‐ Impacts of sharing failures on adaptive management cycle

### 2.1 Distributed decision‐making

Most case studies used top‐down decision‐making systems, which do not allow for the development of locally suitable solutions (Table 1, Figure 2; approach *A*). In contrast, distributed “comanagement” systems—which link governmental institutions with local‐level stakeholders—provide collaborative and flexible learning opportunities that can be adapted as environmental conditions change over time (Berkes, 2009). Encouragingly, the two goose case studies used such systems (Figure 2). In Scotland, a national goose management review group sets the overall agreed national strategy, but local goose management groups have the freedom to set their own objectives and find local solutions. This has allowed contrasting approaches to be developed to suit each system, with sport hunting used as a population reduction tool on Orkney, but government‐led derogation culling used on Islay due to the higher protection status of the goose species concerned (Cusack et al., 2018; Tulloch, Nicol, & Bunnefeld, 2017). The flexibility of this distributed system thus facilitates the establishment of locally suitable and adaptable strategies.

We propose that conflict managers and stakeholders should seek to engage with greater devolution of decision‐making to suit the uniqueness and dynamism of different conflicts. This may not always be straightforward if governance structures are well established, or if existing policy will not allow transfer of powers. Indeed, distributed decision‐making was deemed unfeasible for several case studies (Figure 2), such as with the statutory planning process central to the U.K. bat case study, which may not be possible to devolve over the short term. Where devolution is possible, collaboration needs to happen across management sectors and scales, ensuring that the spatial and temporal needs of conflicts are met and that governance systems fits the characteristics of ecological systems (Guerrero, Bodin, McAllister, & Wilson, 2015). Collaborative governance systems can also have engagement and trust‐building benefits, as has been emphasized recently for conflicts (Bodin, 2017). However, where strong power imbalances exist among stakeholders, there is potential that the initiation of collaborative processes could even exacerbate conflict, for instance, if some groups are unwilling to engage or compromise (Castro & Nielsen, 2001). In such contexts, managers should continually seek to identify and support less represented stakeholders to ensure that collaborative processes facilitate positive change. Further research is required to quantify the trade‐offs associated with devolution for conflict management (Table 2).

### 2.2 Diverse opinions and creativity

No case studies focused on developing innovative management approaches, relying instead on standard conservation methods designed to manage biodiversity and human livelihoods (Figure 2; approach *B*). Reliance on standard (invariably past) practice, designed to meet a single objective, is thought inadequate to balance conflicting stakeholder objectives (Game et al., 2014; Redpath et al., 2013). The payment of compensation to stakeholders impacted by wildlife is a good example of such a conventional approach, being widely used in conflict management (Nyhus, Osofsky, & Ferraro, 2005), including to deal with wolf depredation in Portugal and crop damage by geese on Islay. Such payments can reduce the negative impacts of wildlife but do not address the root causes of conflict, such as divergent values and power imbalances among stakeholders.

We observed that the identified lack of creativity was tightly linked to the restricted range of experts often involved in management (approach *C*). Most case studies relied on specific expertise, such as ecologists (e.g., Orkney geese, Bonaire goats) or practitioners (e.g., planners in Queensland restoration case study; wildlife managers in Iberian wolf case study), rather than combining opinions from a variety of knowledge types and backgrounds (Figure 2). This may restrict the type of interventions that are considered. In contrast, the coproduction of knowledge, particularly by researchers from different disciplines in conjunction with local people, can act as a mechanism for the adaptive learning of management solutions suited to local and dynamic contexts (Armitage, Berkes, Dale, Kocho‐Schellenberg, & Patton, 2011; Tulloch et al., 2017). Such processes were rarely applied in the case studies despite increased knowledge exchange with local communities being a goal of international conventions such as the Convention of Biological Diversity (e.g., Aichi Target 18). An example is seen, however, in the African manatee case study, where management is guided by both knowledge from local fishing communities and evidence from ecologists (Figure 2). Managers here asked fisherfolk to identify key areas for both fishing and manatees, and combined this information with ecological data from manatee activity surveys to identify areas where fishing was most damaging to manatees. By uniting these diverse knowledge types, managers have developed strategies restricting damaging fishing techniques in key areas—such as wide nets deployed across channels—but not from the most profitable fishing zones, thus facilitating the coexistence of fishing and manatees. Developing the networks and actors necessary to coproduce knowledge can be a slow and challenging process (Armitage et al., 2011). However, embracing diverse voices in this way could form an important route for conflict managers to foster creativity. Research into the links between knowledge coproduction, creativity, and conflict is required to fully understand the potential value of diverse voices (Table 2).

### 2.3 Pattern‐based evidence and predictive management

All case studies used passive management, without predicting potential impacts of management on their complex and dynamic environments (Figure 2; approach *D*). Often, this management was labeled “adaptive” (e.g., Islay geese, Orkney geese, and African manatee), as interventions could be adapted over time depending on the response of the system, e.g., adjusting hunting bags depending on population size. However, only true active adaptive management, requiring iterations of scenario‐based predictions and interventions, can tease apart which actions trigger which responses in complex systems (Bunnefeld et al., 2017; Game et al., 2014; Tulloch et al., 2017). Promisingly, an active adaptive decision‐making tool is being developed for the Queensland restoration case study. This optimization software enables managers to predict the iterative effects of interventions on environmental attributes representing their desired objectives (e.g., rate of habitat recovery) under different scenarios. Users can adapt interventions incrementally as new information becomes available each year, identifying appropriate long‐term strategies for achieving specific conflict management objectives in complex and uncertain environments.

Managers could incorporate pattern‐based evidence (Figure 2; approach *E*) into predictive management frameworks by analyzing pattern recurrence in time series of environmental and management variables. Such analyses could pinpoint the environmental conditions under which conflict emerges, and could be particularly beneficial in environments where a number of interacting and uncertain processes influence human–wildlife interactions (Cusack et al., 2018; Mason, Keane, Redpath, & Bunnefeld, 2018). For example, on Islay, goose numbers have increased from 20,000 to >40,000 over 30 years due to interactions between climate change and anthropogenic habitat change converting low‐quality natural habitat into high‐quality grassland (Mason et al., 2018). The growing goose population creates more damage on agricultural land and extreme climate events exacerbate agricultural losses. This becomes a complex system to manage because of tipping points where local people lose income from high‐quality grassland due to damage from geese and climate change. This newly established pattern‐based evidence obtained from 30‐year time series data (Mason et al., 2018) paired with active adaptive management moves this case study forward toward mitigation of conflict.

The types of patterns that managers need to explore may not be environmental patterns typically thought of; for example, identifying the sets of actions by different stakeholders resulting in illegal damage to bat roosts could reveal commonalities in administrative systems that require fixing (U.K. bats). A typical pattern of events resulting in bat roost damage might be for a homeowner to ignore the advice of an architect to request a bat survey, the local planning authority to inadequately screen their planning application, the homeowner to destroy any roost prior to the visit of the planning authority, and an application to be subsequently approved. Analysis of such patterns could reveal how the likelihood of damage could be reduced; here, this might be requiring ecologists to review applications. Managers should expand their view of pattern recognition to encompass not only patterns in ecological dynamics, but also in human behavior and human–wildlife interactions. This could include interactions among stakeholders, such as the alienation of certain groups, which may act as triggers for conflict emergence (e.g., Veríssimo & Campbell, 2015).

There are inherent challenges to implementing truly adaptive management of conservation conflicts. In several case studies, we identified factors that might constrain the use of pattern‐based evidence in predictive management, such as insufficient funding (Iberian wolves, U.K. bats, and Bonaire goats) or data (Iberian wolves) to develop the predictive modeling framework (Figure 2; approach *D*). More broadly, the implementation of such tools also relies to some extent upon adaptive governance systems—flexible comanagement systems of varied actors—capable of implementing the responses suggested by predictive models rapidly and adaptively to changing environmental conditions (Folke et al., 2016). Despite this, we propose that the development of techniques linking active adaptive management and pattern‐based evidence should become a priority area of conservation conflict research (Table 2). Such approaches could lead to substantial improvements in the predictive accuracy of adaptive management simulations, using widely available data sources.

### 2.4 Trade‐off‐based objectives

Maximizing success for one stakeholder group is likely to come at the expense of another (Balint et al., 2011), potentially increasing the perception that one party is asserting its actions over others and exacerbating conflict. For example, restricting where goats can graze on Bonaire shifts the balance toward conservationists, but away from farmers. Measures of success tended to be skewed toward management actions rather than outcomes (Figure 2; approach *F*) and to single‐system components, without acknowledging the diversity of stakeholder views (Figure 2; approach *G*). They were related to conservation (e.g., for African manatee conflict, reducing the number of manatee deaths in fishing nets), livelihoods and the economy (e.g., for U.K. bat conflict, maximizing the number of approved planning applications), or simply project effort (e.g., for Bonaire goats conflict, maximizing the number of goats removed from the natural ecosystem).

Acknowledging that management faces trade‐offs could clarify the strategies most likely to achieve satisficing outcomes. In the Queensland restoration case study, there is a focus on flexibility in how objectives are achieved and variation in stakeholder objectives, following the adoption of structured decision‐making (Guerrero et al., 2017). This decision‐making framework focuses on fundamental objectives, e.g., maximizing persistence of threatened species, meaning that strategies are not “locked in” and a broader range of management alternatives can be considered in subsequent iterations. It also uses stakeholder‐wide consultation to capture a broad range of stakeholder values (e.g., public, NGOs, and industry). Structured decision‐making thus offers a framework that can address the ambiguity and biases in objective setting and should be developed further for conservation conflicts (see also Bunnefeld et al., 2017).

Despite trade‐offs being an inherent feature of conservation, they tend to only be considered while setting out the vision of programs (i.e., governance), rather than operationalizing this vision (i.e., management) (e.g., Boyle, Kay, & Pond, 2001). Further effort is needed to implement management explicitly guided by metrics based on trade‐offs between stakeholder objectives (e.g., Williams, Shoo, Wilson, & Beyer, 2017). A first step toward this goal would be to simulate how conflict dynamics are influenced by objectives based on a single group's interests versus those guided by trade‐offs (Table 2).

### 2.5 Sharing failures

No case studies shared failures transparently (Figure 2; approach *H*). There was a general perception that communicating failed management interventions to the government, general public, or other stakeholders might result in reduced funding or support from important groups. Sharing failures was classified as unfeasible for several case studies, due to the potential for a program to be perceived as ineffective (Figure 2). Particular concerns were losses in future funding (Bonaire goats) and development of mistrust in managers by stakeholders (Bonaire goats, Orkney geese, and Queensland restoration). Wicked problem thinking acknowledges that failures are inevitable, due to the complexities of socioecological systems, and that communicating these openly can optimize management (Game et al., 2014). This echoes with the long‐held view of many conservation practitioners that a “safe‐fail” environment is needed in which practitioners can innovate, experiment, and document failures for others to learn from (Redford & Taber, 2000). However, it has proved highly challenging to foster such an open environment as it requires cooperation between managers and funders, both of whom are under pressure to report only successes. It may be possible to encourage open communication by requiring different parties to formally commit to sharing the risks of projects and to viewing problems not as failures, but as transient features of system interactions (Lloyd‐Walker, Mills, & Walker, 2014). While it may not be possible to fully develop such “no‐blame” cultures within all funding systems, honest discussions between managers and stakeholders about failures—and the potential to learn from them—would provide an important step forward. Little is known of how open communication of failures might feed into the adaptive management cycle; analyses investigating these links are needed (Table 2).

## 3 TOWARD A WICKED CONFLICT APPROACH

Recent studies have developed management approaches and decision‐making frameworks suited to tackling wicked problems in conservation (DeFries & Nagendra, 2017; Game et al., 2014). Here, we take this agenda a step further for a specific conservation problem—conflict—by evaluating if wicked problems approaches are currently being implemented and exploring how a holistic wicked problems strategy could be achieved. We developed five key themes, drawing on varied existing concepts and methods, to deal with different aspects of wickedness. Our themes provide potential transdisciplinary routes toward holistic conflict management strategies and highlight emergent research topics requiring further study (Table 2). Many of these topics could be tackled by meta‐analysis of existing conflicts or by simulating active adaptive management using socioecological models (Bunnefeld et al., 2017).

Wicked problem thinking focuses on tackling systems as a whole, accounting for the complex interactions occurring between social, ecological, and economic elements (Balint et al., 2011). Recent developments in conservation conflict research have concentrated on managing relationships between the people involved in conflicts more effectively, such as developing trust (Mishra, Young, Fiechter, Rutherford, & Redpath, 2017; Young et al., 2016). There is much potential for synergy between these areas, which could set the stage for positive outcomes as trust is built and innovation sparked (Young et al., 2010). For example, the effectiveness of certain wicked approaches, such as embracing diverse opinions and sharing failures, could be enhanced by applying collaborative conservation conflict techniques that build trust and empower local people (Mishra et al., 2017; Young et al., 2016). Approaching conservation challenges holistically is viewed as the way forward for effective conservation in the modern era; a wicked problems approach to conflict can provide an important step toward this.

## Supporting information


**Table S1**. Background information on the spatial scale, principal stakeholders, and primary drivers of eight case studies of conservation conflict
**Table S2**. Description of the characteristics of wicked problems, adapted from Rittel and Webber (1973)
