# Supplementary material for: Wicked conflict: Using wicked problem thinking for holistic management of conservation conflict
Source: Conserv Lett. 2018 Apr 24;11(6):e12460. doi: 10.1111/conl.12460 (PMC6360488; doi:10.1111/conl.12460)
Supplement: Supplementary file 1 — Table S1. Background information on the spatial scale, principal stakeholders, and primary drivers of eight case studies of conservation conflict Table S2. Description of the characteristics of wicked problems, adapted from Rittel and Webber (1973) [file CONL-11-na-s001.docx]

**Supporting Information**

**Table S1.** Background information on the spatial scale, principal stakeholders and primary drivers of eight case studies of conservation conflict.

| **Case study** | **Spatial scale** | **Principal stakeholders** | **Primary drivers of conflict** |
| --- | --- | --- | --- |
| 1. Iberian wolf conservation and farming | Regional | Farmers Hunters Government | Disagreement over the relative importance of wolf conservation and management in the region |
| 2. Barnacle goose conservation and farming | Local/National | Farmers’ groups Conservation NGOs Government organisation | Disagreement over how many geese there should be and how they should be managed |
| 3. Greylag goose conservation and farming | Local/National | Farmers’ groups Conservation NGOs Government organisation | Disagreement over the protected status of geese and who holds responsibility for goose management |
| 4. Bat conservation and planning | National | Local Planning Authority Developers Public (i.e., householders) Statutory consultees | Disagreement over the implementation of legislation to protect bats roosting in development sites |
| 5. Dry forest conservation and goat farming | Local | Goat farmers Conservation NGOs Island government  Dutch government | Disagreement over the extent of damage by goats  to the dry-forest. Disconnect between the people bearing the costs of management (farmers) and those reaping rewards (conservation NGOs) |
| 6. African manatee conservation and fishing | Local | Government agencies Local fisherfolk association Conservation NGOs Traditional leaders | Disagreement over the spatial extent of manatee conservation activities and fishing zones |
| 7. Wetlands conservation and farming | Local/Regional | Farmers Conservationists Government organisation | The lack of management options available to deal with the problem of crop raiding by wetland birds |
| 8. Ecological restoration and planning | Regional | Conservation planners Restoration planners  Restoration practitioners | Disagreement over vegetation restoration objectives (e.g. recovery of habitat for endangered species, maximise visual amenity, improve flood protection) and timeframes for the delivery of outcomes |

**Table S2.** Description of the characteristics of wicked problems, adapted from Rittel and Webber (1973).

|  | **Characteristic** | **Description** |
| --- | --- | --- |
| *Stakeholder* | 1. Conflicting values | Different stakeholders have conflicting values |
|  | 2. Conflicting problem statements | Conflicting values lead to a lack of clarity in problem definition |
|  | 3. Conflicting objectives | Lack of clarity in problem definition leads to confused objectives |
|  | 4. Multiple tactics | Confusion over objectives leads to ambiguity in how to proceed |
|  | 5. Power asymmetry | Different stakeholders have different levels of decision-making power |
| *System* | 6. Dynamic context | The problem is continually evolving |
|  | 7. Scientific complexity and uncertainty | Knowledge of the system is uncertain and incomplete |
|  | 8. Political complexity and uncertainty | Knowledge of the political and power structure is uncertain and incomplete |
|  | 9. Administrative complexity and uncertainty | Knowledge of budget and procedural continuity is uncertain and incomplete |
